# Causes of futile care from the perspective of intensive care unit nurses (I.C.U): qualitative content analysis

**DOI:** 10.1186/s12912-022-01004-y

**Published:** 2022-08-12

**Authors:** Mahnaz Rakhshan, Soodabeh Joolaee, Noushin Mousazadeh, Hamideh Hakimi, Samaneh Bagherian

**Affiliations:** 1 Community Based Psychiatric Care Research Center, School of Nursing and Midwifery, Shiraz University of Medical Sciences, Shiraz, Iran; 2 Department of Evaluation & Research Service, Fraser health authority, Surrey, Canada; 3 Research Center of Health Evaluation & Outcome Science, UBS, Vancouver, Canada; 4 Nursing Care Research Center, Iran University of Medical Science, Tehran, Iran; 5 Department of Nursing, Amol Faculty of Nursing and Midwifery, Mazandaran University of Medical Science, Sari, Iran; 6 Clinical Research Development Unit of Poursina Hospital, Guilan University of Medical Sciences, Rasht, Iran; 7 Department of Operating Room, School of Paramedical Sciences, Birjand University of Medical Sciences, Birjand, Iran

**Keywords:** Futile care, Nurses, Intensive care unit, Qualitative content analysis

## Abstract

**Background:**

Medical care that has therapeutic effects without significant benefits for the patient is called futile care. Intensive Care Units are the most important units in which nurses provide futile care. This study aimed to explain the causes of futile care from the perspective of nurses working in Intensive Care Units are.

**Method:**

The study was conducted using a qualitative approach. Qualitative content analysis was used to analyze the data. Study participants were 17 nurses who were working in the Intensive Care Units are of hospitals in the north of Iran. They were recruited through a purposeful sampling method. Data was gathered using in-depth, semi-structured interviews from March to June 2021. Recruitment was continued until data saturation was reached.

**Results:**

Two main themes, four categories, and thirteen subcategories emerged from the data analysis. The main themes were principlism and caring swamp. The categories were moral foundation, professionalism, compulsory care, and patient’s characteristics.

**Conclusion:**

In general, futile care has challenged nursing staff with complex conflicts. By identifying some of these conflicts, nurses will be able to control such situations and plan for better management strategies. Also, using the findings of this study, nursing managers can adopt supportive strategies to reduce the amount of futile care and thus solve the specific problems of nurses in intensive care units such as burnout, moral stress, and intention to leave.

## Background

Nursing aims to provide quality care and meet the needs of the patient. Now a day, advances in medical methods and technologies have given the healthcare team more opportunities to save patients’ lives. These measures have increased the life expectancy of patients. The growing population of the elderly and the development of new treatments for acute life-threatening conditions have led to an increase in demand for intensive care unit (ICU) beds and the occupancy rate of these beds has become critical [1]. About one is for The United States hospital with intensive care units, recently reported that at least 95% of their I.C.U beds were full. While the national average I.C.U occupancy in 2020 was 67%, according the society of critical care medicine [2] .

Futile care is a situation in which performing life-saving interventions and treatments with significant use of resources has no potential benefit to the patient’s quality of life and cannot end the patient’s dependence on medical care [3, 4]. Futile care refers to invasive treatments or interventions such as resuscitation in end-stage patients, where the probability of successful outcome or patient survival is very low [5–7]. Futile care makes sense in end-of-life patients as well as patients with critical conditions. The intensive care unit is one of the units which is responsible for caring for patients in the later stages of life. Due to the type of patients in these units as well as their urgent need for high-level care, care in these wards faces specific care challenges, such as futile care [8]. Culture, moral values, religion, legal issues, emotional conditions, personal values of the healthcare team members, the patient and the patient’s family, and the patient’s condition affect the use of futile care [9–11]. Deciding whether to start or stop invasive treatments at the end of life is very complicated. In medicine, judging and deciding on the results of the treatment provided to the patient is associated with serious and stressful moral challenges. On the other hand, there are many scientific and ethical aspects, that make decision-making difficult. Physicians and other members of the healthcare team often encounter these situations. Because solving these challenges is so complex, they usually have no choice but to surrender to it and go through it without solving it or taking useful action [12].

Research shows that futile care at the end of life can cause harm to the patient and the moral conflict of the healthcare team [13, 14]. Personnel of intensive care units who provide futile care is at risk of burnout. These situations expose the quality of care and increase staff turnover in these units and indirectly affects the nurses [15–17]. The results of a study showed that caring for patients at the end of life and providing futile care leads to negative experiences and emotions such as frustration, anger, sadness, and guilt in nurses. These issues can cause unbearable pressures and moral distress for the nurse and, as a result, reduce her/him professional satisfaction and eventually leaving the profession [18, 19].

Proving futile care is not cheap. The indefinite duration of benefiting from this care entails great costs for the patient, family, and health system (15–21). The financial loss to hospitals for each patient who needs at least 72 hours of mechanical ventilation is $ 23,000. In addition, there is the cost of insurance and other services. As well as medical centers, families will also suffer from high costs as well as the feeling of helplessness. Eventually, nurses will be hurt due to organizational constraints [20]. Nurses have an important and key role in performing and discontinuing futile care. If they have experience in dealing with such situations, they have a significant and good effect on the patient and the family [21].

In Iran, providing futile care has posed complex challenges to intensive care unit nurses, as well. However, despite the high frequency and complexity of issues related to futile care, little research has been conducted in this regard [19, 21]. Some other Studies have examined nurses’ perception of futile care. For example, Hajiloo and Torabi (2020), Rostami et al. (2017) and Mohammadi et al. (2014) showed that nurses’ have a moderate perception of futile care [6].

In the study of Rezaei et al., the mean score of nurses’ perception of futile care was higher than physicians [22]. Borhani et al. (2015) reported that there is a positive relationship between moral stress and nurses’ perception of futile care [23] .

Although different definitions of futile care have been proposed, there are many ambiguities about it, which have disadvantaged its clarity and clinical use benefits [9, 24]. This ambiguity can lead to incorrect application of this concept in the nursing profession [25]. The existence of ambiguity and complexity in the concept as well as its multidimensionality shows the need to identify and explain this concept in the field of nursing [9] To identify the challenges associated with this issue, especially due to the dependence of this concept on the values, culture, religion, and conditions in Iran, Understanding the experiences of nurses in intensive care units is a key and fundamental step in providing solutions to improve the quality of nursing care in these units. In this regard, the qualitative approach leads us to identify nurses’ perception of situations where they feel the care is futile. The importance of this issue is that the nurse who is in direct and constant contact with the patient can improve the treatment, and connect other specialties of the treatment team to each other and to the patient as well. Therefore, the nurses’ decision in a situation where they feel that care is futile affects the patient’s continued treatment. Quantitative measurement of the nurses’ perceptions of futile care cannot identify the situation that created this perception. So, doing a qualitative research provides an opportunity to answer questions centered on social experience. They are based on the assumption that it is impossible to acquire knowledge about human beings without describing and explaining their beliefs and perceptions in their cultural and social contexts [7]. Therefore, considering the importance of futile care and the necessity of recognizing the reasons for its use in the culture and context of the Iranian nursing community, the present study was conducted to explain the causes of futile care in ICU nurses of hospitals in northern Iran.

## Methods

### Study design

Considering the research question, a qualitative research methodology with a content analysis approach was used. The content analysis approach is a systematic and purposeful way to describe a phenomenon. This approach has been chosen to collect rich and new data [26].

### Participants and setting

The participants included 17 nurses who work in the internal, neurosurgery, poisoning, and general surgery ICUs of hospitals in northern Iran. Only one of the nurses refused to participate in the study. Participants were selected by the purposive sampling method and invited to participate in the study. Inclusion criteria were having at least a bachelor’s degree in nursing, at least 1 year of work experience as a nurse in the ICU, the ability to provide rich information about the concept, and having the appropriate mental and physical condition for the interview. Exclusion criteria included unwillingness to continue the research in subsequent sessions and having an unfavorable mental and physical condition.

### Data collection

The method of data collection in the present study was semi-structured interviews that were done in person or via Skype. The time and type of interview (in person or via Skype) were determined with the consent of the participants. The second author (N.M.) arranged and conducted all the interviews. All interviews were recorded with the permission of the participants. The interviews lasted between 20 and 55 minutes. Interview questions were: What is your understanding of futile care in the ICU? What factors contribute to the futile care in the ICU? Why do they contribute? As the interview continues, follow-up questions such as “Can you explain more? What do you mean? Explain what you mean with an example from your personal experience. Why? And how?” were used to achieve the objectives of the study and based on the response of the participants, and to clarify their responses. The interviews continued until data saturation and no new data was obtained.

### Data analysis

Data were analyzed using Granheim and Landmann’s conventional content analysis approach [27]. All interviews were transcribed. Each transcript was assigned with a number, and identifiers were removed from the transcripts. In the next step, to get a general understanding of the content, the whole text was read several times. Then the meaning units were determined and the initial codes were extracted. The codes were then classified based on their similarities and differences, and finally, the latent content was extracted.

### Study rigor

Prolonged engagement with the phenomenon, devoting sufficient time to data collection, and continuous review of data were used to increase the credibility. The researcher established a good relationship with the participants from the beginning. To ensure the accuracy of the findings and their relevance to the transcribed interviews, some of the interviews were reviewed by three professors of Nursing (peer review) who are experts in qualitative and nursing research along with the codes and categories. Interviews and codes were also presented to the four participants in the study, who stated that the findings were consistent with their understanding and interpretation. The researcher reduced the likelihood of bias in collecting, analyzing, and coding participants’ statements and improved the validity of the data by limiting the literature review at the beginning of the study. To achieve credibility, all stages of the work were independently reviewed by external reviewers (qualitative researchers) and then the results were compared. To reach confirmability, all steps of data collection and analysis were described and audit step by step. While qualitative researches do not have generalizability, we ensured transferability by providing clear descriptions of the selection of participants, data collection, work process, and research data [28].

### Ethical considerations

The project has been approved by the Vice-Chancellor for Research of Mazandaran University of Medical Sciences (IR. MAZUMS.REC.1400.11547). Before each interview, the researcher explained the purpose of the study to the participants. They were reassured that their information will be confidential and their participation is voluntary. Informed written consent was obtained from them to participate in the study and recording of audio. The place and time of the interview were also determined based on participants’ will. Their anonymity was maintained at all stages of the research.

## Results

Participants in the study were seventeen nurses working in the ICUs from six hospitals in the northern provinces of Iran. Most of the participants were women (70.59%) and married (52.95%). Four of them had a master’s degree (23.53%), one had a Ph.D. (5.89%), one had an associate degree (5.89%), and the rest had bachelor’s degrees (64.69%). The age range of participants was between 24 and 50 years and they had 1–27 years of work experience. Table 1 shows the demographic characteristics of the participants. From the data analysis, 789 primary codes, two themes entitled principlism and care swamp, four categories, and thirteen sub-categories were extracted. Classes included basics of ethics, professionalism, compulsory care, and patient characteristics. (Table 2).

**Table 1:** Demographic characteristics of participants

participant	Gender	Age	Work experience	Work experience In I.C.U	Marital status	Educational Status
1	Male	40	10	5	Married	BSc
2	Female	32	13	4	Married	Associate degree
3	Female	27	3	3	Single	BSc
4	Female	36	12	8	Single	Phd student
5	Female	42	16	10	Single	MSc
6	Female	48	20	12	Married	MSc
7	Female	24	1	1	Married	BSc
8	Female	45	15	8	Single	BSc
9	Male	30	5	3	Married	BSc
10	Female	42	13	8	Single	MSc
11	Female	33	7	2	Single	BSc
12	Female	37	12	5	Married	BSc
13	Male	36	10	6	Married	BSc
14	Female	34	12	4	Single	BSc
15	Male	25	4	3	Married	BSc
16	Female	50	27	9	Single	MSc
17	Male	46	16	10	Married	BSc

**Table 2:** Themes, categories, and subcategories of causes of futile care from the perspective of nurses

Theme	Categor	Subcategory
principlism	Moral foundation	Family Background
		Religion beliefs
		Conscience
	Professionalism	Philosophy of nursing
		Altruism
		Human Dignity
Care Swamp	Compulsory care	Lack of the same instructions
		Fear of law
		Fear of patient’s family
		Fear of authorities
		Family insistence
	Patient’s characteristic	Patient’s age
		Role of the patient in the family

### Principlism

principlism was one of the themes extracted from the interviews. In the nursing theory, principlism refers to care based on predetermined principles, which can be a criterion for evaluating the provided care [29]. The categories of this theme were Moral foundation and Professionalism.

### Moral foundation

Moral is a set of do’s and don’ts that are inseparable parts of people’s lives and give them meaning and direction. In fact, there is no human activity that does not require morality during life, then, morality must be evident in all behaviors and actions of individuals.

The moral values of the individual, which are formed following education in the family and society and mixed with the religious beliefs of the individual and internalized, affect the provision of futile care. The family transmits moral values to children, which has a special role in strengthening the person’s moral character. In this regard, participant 7 said:

“ … Based on my family upbringing, I have learned to help as much as I can, so I take care of my patient as much as I can.”

Most participants said that they consider religious principles during care provision. In this regard, participant 10 said:

“I believe that there is a God who gives life and takes it and I am a tool and as long as the patient is alive, I will do my best.”

Internal conscience is another sub-category of moral foundation that was extracted from data analysis.

Conscience is the inner force in the mind, which anyone uses to decide about the good and bad of behaviors. Conscience is a readiness, insight, or judgment that helps to distinguish right from wrong. Judging behaviors based on values or norms is called conscience [30].

“When I am in these situations, it is like something inside me is telling me to do my job, it seems I am making a decision based on my heart at that moment. Logically, I should not do this, but my conscience tells me to do it.” (Participant 1)

### Professionalism

Professionalism is one of the basic concepts of nursing and care. Professionalism is very important in the ethical path of the employees of a profession. The slightest sign or indication of the unprofessional performance of nurses or their indifference in the performance of services, can lead to harms to people.

Professionalism addresses ethical issues and questions, as well as the ethical principles and values of a profession such as nursing. According to the nurses, the philosophy of nursing, altruism, and respecting human dignity are among the things that can show their professionalism. Philosophy is a set of principles and ideas about a phenomenon. The philosophy of nursing includes the nature of nursing, and its ontology, epistemology, and ethics, which produces professional ideas and theories [31] .

“We have been told since we became nursing students that you should take care of your patient in any situation. Our teachers educated us that your discipline is different from other disciplines. The nature of our discipline is different.” (Participant 6)

The next subcategory was altruism.

“When I have an end-stage patient, my desire for help increases. I worry about her/him more. I want to do more for her/him so that she/he gets a little better. I do not want any reward from anyone. The patient is a human, like me who needs me more at that moment.” (Participant 11)

Human dignity is another sub-category of professionalism that was extracted from data analysis. This means that human beings, regardless of race or gender, deserve respect.

“When I see that my patient is in a very bad condition, for example, he is bleeding, he has a lot of pulmonary secretions, he has respiratory distress, or he is in a very bad situation, I really cannot do nothing because I think people deserve special services because of their dignity. Medical services and nursing.” (Participant 3)

Considering the Islamic nature of Iranian society and the impact of religion and ethics on various aspects of people’s lives, as well as the existence of altruism, valuing their dignity and adhering to the philosophy of nursing can be an important factor in providing futile care from the nurses’ point of view. The significance reveals particularly when all care is done and there is no hope for the treatment and recovery of patients.

### Care swamp

Care swamp is another theme that emerged from the interviews. It included two categories: compulsory care and patient characteristics.

### Compulsory care

The results of analyzing the data obtained from the participants showed that nurses sometimes have to take some care due to the existing conditions. Participants stated that lack of the consistent instruction, especially when dealing with patients in the end stages of life for whom there is no hope of treatment, fear of law, fear of the patient’s family, fear of authorities, and in some cases, family’s insistence on continuing treatments despite their ineffectiveness requires them to provide care, sometimes ineffectively and compulsorily**.**

The results of analyzing the data obtained from the participants showed that nurses sometimes have to provide care due to the existing conditions.

The inconsistency of instructions in the face of futile care is one of the sub-categories resulting from data analysis.

“Swore to God, we still do not understand what to do when we are in such situations. Because it seems that there is no single instruction in this regard and everyone acts as his or her wish. “(Participant 17)

Fear of the law was mentioned as another factor in nurses’ futile care.

“I am afraid of the legal consequences of not doing work for the patient. Honestly, if there was a law about not doing futile care, I would act based on that. Then, as long as there is no law in this regard, I will do my job to the end, because I do not have the tolerance of facing the court and the law.” (participant 9)

Fear of the patient relatives is another cause of futile care that was mentioned by the nurses.

“If I have an end-stage patient and I know he/she does not have a good prognosis. And I ignore some of the care. If the family of the patient finds out about that they will make trouble.” (Participant 12)

Participating nurses acknowledged that fear of the authorities was another important factor in providing futile care.

“Once I had a patient whose level of consciousness was five. He was arrested and we started resuscitation. However, because we knew he had no good prognosis, we did not try very hard. The next day when our head nurse came and found out, she had taken me to a corner and questioned me.” (Participant 8)

The insistence of the families forces the nurse to provide care while she knows that the care is futile.

“Most of the time we know that it is better not to do anything for the patient. However, the patient's family is so insistent on keeping the patient alive that we also have to keep the patient alive in any way possible. “(Participant 16)

### Patients characteristics

The last extracted category is patient characteristics with has two sub-categories: patient age and patient role in the family.

The patient’s age and role in their family affect the nurse’s emotions and force them to provide futile care.

The child or adolescent patient leads to the nurses’ emotional arousal and a factor for futile care. Also, if the patient plays key roles in the family like father, mother or head of the family, the need for care, even futile care increases.

“If I see that the patient is very young, I will do all the care until the end.” (Participant 11)

“If I see that the patient is the father of the family or, for example, he is the head of the family, then futile care does not make sense to me, and as long as his heart is working, I will provide my treatment and care.” (Participant 15)

There is a lack of the same policy on how to care for critically ill patients who are in the final stages of their lives and who sometimes suffer a lot. The legal problems that nurses may face in many cases, the fear of reprimand from the authorities, the concern of the patient’s companions, and their insistence on continuing care are serious factors in providing care in vain. The role that the patient has in the family, which can be the role of father and provider of livelihood or the role of mother, as well as young patients, are also among the factors that lead nurses to provide futile care.

## Discussion

This study aimed to explain the causes of futile care from the perspective of nurses working in the ICU. Two themes, four categories, and thirteen subcategories emerged from data analysis. The main themes were principlism and caring swamp. The categories were moral foundation, professionalism, compulsory care, and patient’s characteristics.

One of the issues that have been raised in this study from the participant’s point of view as a cause of futile care is the moral foundations. The moral values of the individual, which are formed in the family and society and are mixed with the religious beliefs of the individual can influence the futile care. Some nurses see their personal and cultural beliefs as a motivator to provide futile care [11]. In the study of Moaddaby et al. (2021), which examined the perception of Futile care and its relationship with moral distress in ICU nurses, individual beliefs and values had the highest mean score of reasons for providing futile care [32]. The results of the study conducted by YekeFalah (2018), which examined the causes of futile care in end of life patients in the ICU from the perspective of nurses, showed that the rarest reasons for futile care were related to the dimensions of nurses’ professional competence and personal beliefs and values [11]. Differences in nurses’ perceptions in different studies can be due to cultural differences, the use of different study tools, and differences in design and study subject. Alazmani-Noodeh (2021) study showed that spiritual beliefs have a moderating effect on the relationship between futile care and job satisfaction of nurses [9]. In Rafii’s (2020) study one of the concepts includes the values ​​and beliefs of the health care team about the definition of futile care which expresses the results of fieldwork, including the implementation of all instructions and the need for all evidence-based interventions [33] . Since the findings of the present study are consistent with YekehFallah and Rafii conducted on the Iranian nursing community, it seems that the values ​​and beliefs of nurses are effective in futile care [11, 33].

Another factor that, from the participants’ point of view, has been suggested as the cause of futile care was professionalism. According to the nurses, the philosophy of nursing, altruism, and respecting human dignity are the items that can show their professionalism. Participants considered care as the duty and nature of the nursing profession and emphasized that caring is the soul and essence of nursing work.

In the study of Aghabarari (2019), the participants believed that the outcome of care should be the patient’s recovery. However, in cases where the patient’s recovery is not possible for some reason, we can provide relief and comfort, maintain the patient’s dignity, and increase his/her quality of life by considering the mental, psychological and spiritual along with the physical care according to patient’s needs. Even in the last days of his/her life [8]. In the study of Yekeh Fallah (2018), in terms of professional competence, non-observance of ethical principles by physicians in dealing with patients’ families and not telling the truth and obtaining compulsory consent to perform various procedures were among the important reasons for increasing futile care in the ICU. Giving false hopes to the family and rejecting to telling the truth by physicians lead to a reduction in the number of patients being transferred home and an increase in the length of their stay in the ICU [11]. According to the results of other studies, one of the reasons for physicians’ unwillingness to remove futile care is the desire to satisfy the patient’s family (31, 32). In the study of Bahramnejad et al. (2019) who have defined and clarified the concept of futile care, they named some reasons such as unclear mental, psychological and biological dimensions of individuals, the existence of ethical issues, respect for maintaining the patient’s independence in decision making [34].

The results of analyzing the data obtained from the participants showed that nurses sometimes have to provide care due to the existing conditions. The inconsistency of instructions in the face of futile care is one of the sub-categories resulting from data analysis. In the study of Yekeh Fallah (2018), in terms of organizational policy, the lack of a committee to decide on the transfer of dying patients to his/her home or another ward was mentioned as the most common reason for futile care. Also, the most common reason for futile care in terms of legal issues was the lack of national laws or unity of procedure regarding hospitalization and treatment of dying patients [11]. In the study of Bahramnejad et al. (2019), the lack of clear instructions about futile care and what care is called futile, and the lack of a boundary between futile and fruitful care were mentioned [34].

Jox et al. (2012) also believe that the lack of explicit instructions for nurses regarding futile care is one of the main problems of nurses when they face such care [35]. The lack of guidelines for nurses about futile care is one of the main problems for them in this situation [36]. Therefore, to reduce the futile care and its effects on nurses, some measures should be taken to remove the existing barriers and improve the necessary facilities by authorities.

Most of the participants in our study reported that Fear of the law, the authorities, and the patient’s family was also mentioned as other factors for nurses in providing futile care. Many participants also felt that physicians were the driving force behind futile care, especially when there was disagreement among team members about the futility of care.

Bahramnejad et al. (2019) in their study also mentioned the fear of legal pressures, unrealistic expectations of the family from clinical caregivers, the physician’s fear of distrust of the family, and the fact that the family does not want to follow the patient’s offer of no treatment as reasons for providing futile care [34]. The results of Courteright et al.’s showed that while there is no history of suing the health care team for not doing futile CPR, the fear of litigation is the reason for continuing CPR despite the belief that it is not fruitful [37]. According to the results of Hollander et al. and Vergano, the causes of futile care are the ambiguity of hospital policies and the fear of litigation [38, 39]. The participants stated that families’ struggles and insistence force them to provide futile care. Santonocito et al. believe that despite the treatment team’s desire to doing the CPR when a patient requests no CPR, the patient’s request and his/her independence should be considered and the patient should be allowed to die respectfully. However, there is currently a real challenge between the patient and the family with physicians, nurses, and the community in this regard [40]. Disagreement over situations in need of resuscitation is one of the most common sources of conflict in the clinic [36]. The study of Baljani et al. (2012) showed that physicians’ ignorance regarding the patient and his/her family requests and making decisions without informing them leads to an increase in the amount of futile care [41]. The Sibbald study also noted that healthcare members were pressured by family members to provide futile care [42].

It seems that there are no clear guidelines for determining the limits and examples of futile care, especially in Iran; drawing up a line of specific procedures for decision-making in futile care, particularly in cases such as end-of-life stages, providing care seems essential. Due to the moral stress in physicians and nurses caused by futile care, they need to undergo comprehensive training courses on ethical principles related to futile care. In addition, since improving the quality of care cannot be achieved without the support and various cultural and legal contexts, creating awareness in society, especially for the families of patients in special wards are also necessary for problems arising from futile care.

Participants also stated that the patient’s age and role in the family affect the nurse’s emotions and force them to provide futile care. They said that if the patient is young or the head of the family, futile care does not make sense and they will continue their treatment as long as the patient’s heart is working.

The definition of futile care varies according to patients’ conditions and nurses’ values [21]. Participants also stated that the patient’s age and his/her role in the family affect the nurses’ emotions and force them to provide futile care. Nurses who are working in intensive care units often deal with futile care for weeks or months. Because after 12 hours of physical and emotional care of patients, nurses become emotionally dependent on them and these nurses are exposed to moral stress due to the provision of futile care [17].

### Limitations

Although the findings of the present study help explain the causes of nurses’ futile care, there are some limitations. For example, due to the Covid-19 epidemic, it was not possible to perform focus group discussions to generate interactive data. Also, the high workload due to lack of staff and also the lack of comprehensive support for nurses in intensive care units was another limitation of the present study that could affect the response of participants. We tried to attract the cooperation of nurses by explaining more about the goals of the project and its usefulness, or an interview was conducted at another time. Also, the mental state of the participants could affect the data. This limitation was reduced by determining the time of the interview according to the participant’s wish. Another limitation of the present study is that this study examined the perspectives of nurses regarding the causes of futile care, while. We recommend investigating these causes according to the opinions of all members of the treatment team, in future studies. Finally, this study was conducted in hospitals in northern Iran and the adult ICU which can be another limitation. We recommend that future studies can be conducted in hospitals in other parts of Iran, as well as in the ICU of children and infants.

## Conclusion

Principlism and adherence to principles, along with the care swamp, cause nurses to get stuck in a sequence that complicates the fruitiness of care. In general, futile care has confronted nursing staff with complex conflicts. By identifying some of these conflicts, nurses will be able to manage such situations and plan for better management. Since nurses can play a key role in the management of futile care, knowing the causes of futile care can be an important step in developing useful care programs in ICUs.

Doing futile care exposes nurses to burnout, frustration, anger, and moral distress, which in turn can affect the quality of their work. Providing end-of-life care for patients with no hope of survival, is challenging. Prescribing rare drugs and performing various diagnostic procedures, which in some cases can be painful, increase the patients’ problems such as long hospital stay, increased workload of nurses, increased expenses for families and the health care system. As a result, the findings of the present study can be a step towards the development of guidelines and care policies for end-of-life patient in ICUs. As a result, some patients who are in the final stage of their illness, can receive hospice care. In this way, the capacity of ICU beds for patients in need can be increased.

Nursing managers can adopt supportive strategies based on our findings to reduce the amount of futile care and solve the problems of nurses in intensive unit’s wards such as burnout, moral stress, and intention to leave. There are no instructions in Iran that clarify the limits and examples of futile care. Therefore, it seems necessary to develop guidelines for deciding on futile care, especially in cases such as end-of-life care.

## Data Availability

The data that support the findings of this study are available from corresponding author but restrictions apply to the availability of these data, which were used under license for the current study, and so are not publicly available.

## Abbreviation

ICU: Intensive care unit
